# The critical role of job embeddedness: The impact of psychological empowerment and learning orientation on organizational commitment

**DOI:** 10.3389/fpsyg.2022.1014186

**Published:** 2022-12-05

**Authors:** Dong-Yeol Yoon, Caleb Seung-Hyun Han, Soo-Kyoung Lee, Jun Cho, Moonju Sung, Soo Jeoung Han

**Affiliations:** ^1^School of Business Administration, Konkuk University, Seoul, South Korea; ^2^Department of Lifelong Education, Administration, and Policy, University of Georgia, Athens, Georgia; ^3^School of Business Administration, Konkuk University, Seoul, South Korea; ^4^Human Resources Development Service of Korea, Ulsan, South Korea; ^5^National Assembly Futures Institute, Seoul, South Korea; ^6^Graduate School of Education, Yonsei University, Seoul, South Korea

**Keywords:** job embeddedness, psychological empowerment, learning orientation, organizational commitment, mediating role

## Abstract

Job embeddedness (JE) has been recognized as a key factor to address the issue of employee turnover and employee attitudes. This study explores underlying mechanisms of job embeddedness that link the organizational environment and the individuals’ perceptions of the job. Particularly, the effects of psychological empowerment and learning orientation on organizational commitment were examined. This study hypothesizes that psychological empowerment (PE) and learning orientation (LO) should influence organizational commitment (OC) and job embeddedness plays a significant mediating role in these relationships. Data were collected from 27 offices of Human Resource Development Service of Korea (governmental agency) located in major cities in South Korea. Results indicate that all hypothesized relationships (PE and JE, LO and JE, LO and OC, JE and OC, and the mediating role of JE) are supported, except for psychological empowerment and organizational commitment. While the impact of psychological empowerment was not significantly related to organizational commitment, it is notable that through job embeddedness, psychological empowerment had indirect effects on organizational commitment. Further, learning orientation had significant effects on job embeddedness and organizational commitment. Lastly, the most compelling finding is a full mediation of job embeddedness in the relationship between psychological empowerment and organization commitment. Implications for research and practice are discussed.

## Introduction

Over the past decade, business environments and job markets have become volatile, and the issue of employee turnover persists as a growing concern for many organizations (Mitchell et al., 2001; Holtom and O’neill, 2004; Felps et al., 2009). Job embeddedness (JE) has been recognized as a key factor to address the issue of employee turnover (Mitchell et al., 2001; Crossley et al., 2007; Allen and Shanock, 2013; Sender et al., 2018). To conceptualize job embeddedness, Mitchell et al. (2001) focused on why employees remain and linked the concept to favorable employee attitudes. Job embeddedness is recognized as an individual level phenomenon, and it is based on a balance between perceived costs and psychological benefits. Job embeddedness is also regarded as a key mediating construct between work-related organizational factors and employee attitudes (Li et al., 2016). However, underlying mechanisms that link the organizational environment and the individuals’ perceptions of the job remains unknown (Kiazad et al., 2015). Therefore, this study attempts to reveal the mechanism of how job embeddedness works between organizations and individuals.

Additionally, our aim is that this study extends extant job embeddedness theory to non-Western countries, such as South Korea, because different value systems in specific countries may impact employees’ perceptions in different ways (Williamson and Holmes, 2015; Jordan et al., 2017). Through literature review on embeddedness, Ghosh and Gurunathan (2015) found that existing studies on embeddedness are mostly restricted to the West; however, studies in Asian countries still remained largely unexplored.

The cultural setting in South Korea makes this study meaningful in the job embeddedness literature. That is, this study examined the mediating effects of job embeddedness in a unique cultural setting of Korean corporations, where an organizational culture of hierarchy, collectivism, and masculinity prevails (Hofstede, 1998). More specifically, organizational cultures in Korea encourage employees’ interdependence with the organization, a larger power distance between leaders and subordinates, and putting more emphasis on “off-the-job” factors to explain organizational commitment, in contrast to the organizational factors in Western society (Shore, 2013). Taking into account the importance of this Korean organizational culture as we interpreted the findings of the current study, as such a collectivistic culture could oftentimes notably put pressure on employees into socially obligated organizational behaviors at the workplace, the design for this study strengthened the linkage between psychological empowerment, learning orientation, and organizational commitment through the perception of job embeddedness. Conducting a similar study in Western countries, which hold a different organizational culture with the emphasis on smaller power distance and individualism within corporations and organizations, would help us better understand the significance of the effect of such an meaningful distinctiveness.

Responding to this gap, this study proposes that perceived employees’ psychological empowerment would play a significant role in the organizational commitment that leads to organizational performance, and job embeddedness will mediate this relationship. Specifically, psychological empowerment refers to “psychological motivation reflecting a sense of self-control in relation to one’s work and an active involvement with one’s work role” (Seibert et al., 2011, p. 981). Psychological empowerment is comprised of multidimensional cognitive factors consisting of meaning, self-determination, competence, and impact. Individuals with a feeling of empowerment possess and can rely on a proactive orientation to one’s work roles (Sun L. Y. et al., 2012). Thereby, we argue that psychological empowerment nourishes individual’s job embeddedness, which in turn improves their attitudes towards their organizations.

Another key construct in the proposed mechanism is learning orientation. When employees appreciate the benefits of growth and opportunities from training and learning, they reciprocate such support in the form of positive attitude (Bunderson and Sutcliffe, 2003). In this regard, an employee’s commitment may be the result of a perception that their interests, through learning and development, are supported. However, few researchers have examined the importance of learning orientation with employees’ job embeddedness (Shah et al., 2020). We assume that opportunity-enhancing learning orientation may improve employees’ perception toward the organization further by embedding them in the job. Our study is cognizant that motivational and environmental factors are likely to affect the way job embeddedness relates to employee’s attitude. We explore the mediating role of job embeddedness among psychological empowerment, learning orientation, and organizational commitment.

Along with job embeddedness theory, studies on employees’ organizational commitment (OC) remain an important construct to explaining talent retention and for developing human resources (HR) in organizations (Kontoghiorghes, 2016; Mathieu et al., 2016). Effectively facilitating employee’s commitment by providing psychological empowerment (PE) and promoting learning orientation (LO) is important for enhancing organizational capacity and capability (Bani et al., 2014). Unfortunately, few studies delineate the motivational and psychological effects that explain the development of organizational commitment.

Therefore, this study had two specific goals. First, we sought to extend job embeddedness theory and research on organizational commitment by demonstrating how job embeddedness bridges a link from psychological empowerment and learning orientation. Second, we sought to determine whether the variables of psychological empowerment and learning orientation are predictors of organizational commitment. These two goals address the needs for examining the critical role of motivation and psychological empowerment noted in the job embeddedness literature.

## Literature review

### Psychological empowerment

Psychological empowerment is one of the widely used contextual variables in management research. Increasingly, researchers examine attitudinal and behavioral outcomes, including job performance, in relation to psychological empowerment (Maynard et al., 2014). Conger and Kanungo (1988) proposed that empowerment be viewed as a motivational construct-meaning to enable rather than simply to delegate. Thomas and Velthouse (1990) also empowerment was conceptualized in terms of changes in cognitive variables which determine motivation in workers.

Spreitzer (1995) operationalized theoretical work by creating a measurement of psychological empowerment and proposed a second-order factor of psychological empowerment. It consisted of four dimensions that combined additively to form an overall construct of psychological empowerment. The four dimensions consisted of (1) meaning—the values, beliefs, and work purpose judged by individual’s ideals, (2) competence—an individual’s efficacy specific to accomplish their work role with skills, (3) self-determination—an individual’s sense of initiatives for work behaviors and processes, and (4) impact—the degree an individual can influence work role outcomes at work.

Several previous studies found that psychological empowerment is positively associated with a variety of outcomes. Example attitudinal consequences of psychological empowerment are higher job satisfaction (Spreitzer et al., 1997), higher organizational commitment (Barroso Castro et al., 2008), a reverse relation to job strain (Harley et al., 2007), and lower turnover intention (Griffeth et al., 2000), employee creativity (Matsuo, 2022). Behavioral consequences are a higher level of task performance (Humphrey et al., 2007; Zada et al., 2022), innovation, and managerial effectiveness (Spreitzer, 1995).

### Learning orientation

Learning orientation in organizational contexts is defined as “organization-wide activity of creating and using knowledge to enhance a firm’s competitive advantage” (Calantone et al., 2002, p. 516). Watkins and Marsick (2019) employ a cultural perspective of organizational learning that promotes learning capacity to transform as a continuous and strategically used process in formal and, especially, informal learning. Aligning with the concept of a learning organization, learning orientation attempts to connect the organization to its external environment (Watkins and Marsick, 1993).

The operationalization of learning orientation consists of (1) commitment to learning, (2) shared vision, and (3) open-mindedness. First, the main piece of learning orientation is the value placed on learning by an organization (Sinkula et al., 1997). Learning orientation emphasizes the primary means of enhanced capacity to learn and grow. Second, shared vision refers to the direction of learning by providing a focus for learning that assists in the understanding of what needs to be learned (Sinkula et al., 1997). Third, open-mindedness reflects the value that an organization proactively questions the past and regards the future with the ability to change. These organizational characteristics capture how the organization can facilitate or influence an individual employee’s organizational behaviors through structure and environmental atmosphere (Baker and Sinkula, 1999).

### Job embeddedness

Job embeddedness refers to “a construct composed of contextual and perceptual forces that bind people to the location, people, and issues at work” (Crossley et al., 2007, p. 1031). The critical aspects of job embeddedness in assessments are internal and external factors that affect individuals’ (a) links to teams and other people; (b) perception of fit with their jobs, organizations, and communities; and (c) likely reactions regarding what they would have to sacrifice if they left their jobs (Mitchell et al., 2001; Kiazad et al., 2015). This last factor was conceived to capture why people remain with the organization against voluntary departure possibilities. Together, these three aspects are labeled as links, fit, and sacrifice on the intricate aspects of community and individual bonds that align with organizational goals and strategies.

### Organizational commitment

Organizational commitment refers to “the relative strength of an individual’s identification with and involvement in a particular organization” (Mowday et al., 1982, p. 27). Organizational commitment is a multi-dimensional construct that denotes the relative strength of an individual’s identification, involvement, and loyalty to a particular organization (Meyer and Allen, 1997). Affective commitment reflects an emotional attachment to the organization based on feelings of loyalty toward the employer. Continuance commitment is based on perceived costs of leaving the organization. Normative commitment means a sense of obligation on the part of the employee’s membership in the organization.

Many empirical studies of affective organizational commitment reported positive relationships with job-related experience and organizational factors as antecedents to organizational commitment (Laschinger et al., 2002; Kim et al., 2016; Karim, 2017). For example, job resources that have positive psychological consequences strengthen organizational commitment (Guenzi and Nijssen, 2021). And supportive HR practices signal organizational concerns for its employees and these signals elicit attitudinal and, presumably, behavioral responses, such as increased commitment, continued service to the organization, and a lower intent to quit which results in lowered actual turnover (Seibert et al., 2011). However, since continuous commitment is related to the leaving cost of employees, it was suggested that the relationship between psychological empowerment and continuous commitment would be low.

#### Psychological empowerment, job embeddedness, and organizational commitment

Avolio et al. (2004) examined psychological empowerment as an antecedent of organizational commitment and as a mediator between transformational leadership and commitment. That study found a positive direct relationship between psychological empowerment and organizational commitment and a significant indirect effect of psychological empowerment. Other studies reported psychological empowerment as a significant antecedent of organizational commitment (Joo and Shim, 2010; Ouyang et al., 2015). Bani et al. (2014) studied the association between psychological empowerment (in terms of sense of efficacy, meaningfulness, autonomy, and trust) and job embeddedness, and they found a positive association between those two constructs. Positive associations between psychological empowerment and job embeddedness were supported from several other studies (Jeon and Yom, 2014; Karavardar, 2014; Bin Jomah, 2017).

Thomas and Velthouse (1990) suggested that empowerment leads to higher levels of initiative and concentration, which in turn increase organizational commitment. Further, Spreitzer (1995) suggested that empowered employees will regard themselves as more capable of managing their work roles in a more meaningful way by forming a higher level of commitment when empowered (Spreitzer, 1995). Seibert et al. (2011) examined a cross-level model of psychological empowerment. They proposed a model with psychological empowerment as a determinant of individual attitudes, particularly job satisfaction and organizational commitment at the individual level. They reported findings that showed psychological empowerment as positively related to both attitudes. Also, Meyer and Allen (1997) noted the role of psychological empowerment as an intrinsic form of motivation in relation to affective commitment. Therefore, we hypothesize:

*H1a*: Psychological empowerment will be positively related to job embeddedness.

*H1b*: Psychological empowerment will be positively related to organizational commitment.

#### Learning orientation, job embeddedness, and organizational commitment

Learning orientation attempts to develop employees who are willing to combine their own personal learning with broader collective action in an organization (Senge, 2014). This learning-oriented approach in organizations has facilitated employees’ job adaptation so that they can perform effectively and creatively (Dweck and Leggett, 1988). Previous empirical studies suggest that learning organizations can facilitate desirable outcomes for both individuals and organizations. For example, scholars found that learning organization affected job embeddedness positively (Kanten et al., 2015). Lee et al. (2013) found that the mediating effect of job embeddedness had a significant effect between learning organization and job satisfaction. Also, Joo and Shim (2010) found a positive influence of learning organization culture toward organizational commitment. Hanaysha (2016) also confirmed that organizational learning has a positive impact on organizational commitment. Therefore, we suggest,

*H2a*: Learning orientation will be positively related to job embeddedness.

*H2b*: Learning orientation will be positively related to organizational commitment.

#### Job embeddedness and organizational commitment

An employee’s organizational commitment is strongly associated with the nature of fit between individuals and their organizations (Mitchell et al., 2001). Several empirical studies stated that a strong level of job embeddedness was associated with effective job performance and low intention to leave (Halbesleben and Wheeler, 2008; Ramesh and Gelfand, 2010). Job embeddedness that used three dimensions (fit, links, and sacrifice), as in this study, predicted not only intent to leave but also other key outcomes, such as organizational commitment and job satisfaction (Mitchell et al., 2001; Kim and Kang, 2015). Kim and Kang (2015) found that both job embeddedness and organizational commitment were identified as most likely paths to turn-over intentions, and those two variables were positively related. Another study found that the stronger the level of job embeddedness, the more links an individual is likely to have and to be committed to the organization (Nica, 2018). In addition, job embeddedness has a significant effect on improving employee well-being, one of the variables that affects organizational commitment (Ahmad et al., 2022). Thus, the aforementioned literature suggests the following hypothesis:

*H3*: Job embeddedness will be positively related to organizational commitment.

### Mediating role of job embeddedness

Retention is a critical concern for many organizations. The most frequent variables used as a predictor for turnover rates are job embeddedness and organizational commitment (Williamson and Holmes, 2015). Regarding job embeddedness, researchers noted that more embedded employees are less likely to voluntarily leave the organization (Mitchell et al., 2001). Several scholars empirically tested the phenomenon by using different variables, such as socialization tactics, organizational support, job embeddedness, organizational commitment, and turnover intentions (Allen and Shanock, 2013). They found that job embeddedness mediated a relationship between socialization tactics (e.g., networking) and job commitment because the more employees feel value in the relationships among employees and belonging, the more they will be satisfied with their work. Frequent social exchange among employees will lead to attitudinal and behavioral commitment by giving a sense of positive relationships (Yoon and Lawler, 2006). Organizations should be proactive about increasing job embeddedness among employees because establishing or increasing job embeddedness is likely to increase retention, attendance, citizenship, and job performance (Mitchell et al., 2001; Lee et al., 2004, 2014).

Scholars examined job embeddedness to answer why employees remain in their organizations. Qian et al. (2022) suggest highly embedded in the organization can help employees less vulnerable to job insecurity. Another of the studies investigated the effects of job embeddedness as a moderator of relationships among leader-member exchanges, organization-based self-esteem, organizational citizen behaviors, and task performance (Sekiguchi et al., 2008). That study found that job embeddedness moderated the relationship between self-esteem and organizational citizenship behaviors. As self-esteem and quality of relationship are similar concepts to psychological empowerment, we expected similar patterns of interactions on the relationship as hypothesized below:

*H4a*: Job embeddedness will mediate the relationship between psychological empowerment and organizational commitment.

*H4b*: Job embeddedness will mediate the relationship between learning orientation and organizational commitment.

## Materials and methods

### Data collection and sample

Data were collected from 27 offices of Human Resource Development Service of Korea (governmental agency) located in major cities in South Korea. All 430 employees involved were contacted by HR directors and received a written questionnaire along with a cover letter asking for their confidentiality and voluntary participation in this study. The survey was administrated by randomly assigned identification numbers. A total of 391 employees (91%) completed and returned the survey. Also, 48 sets of missing data were deleted based on list-wise deletion. The sample was 27.1% female and 72.9% male, which shows a very male-dominated organization. Half of the participants (44.6%) were aged in their 30s and 24.5% in their 20s. Over half of the respondents held a bachelor’s degree. There were no significant differences of responses in gender and age (see Table 1).

**Table 1:** Sample demographic data (*N* = 343).

	Values	Frequency	Percentage
Gender	Male	250	72.9
	Female	93	27.1
Age	20s	84	24.5
	30s	153	44.6
	40s	78	22.7
	50s	28	8.2
Education	High school	78	22.7
	Bachelor	215	62.7
	Graduate	50	14.6
Position	Staff	136	39.7
	Assistant manager	91	26.5
	General manager	41	12.0
	Senior manager	46	13.4
	Director	29	8.5
Years of work	Less than 1 years	107	31.2
	1–5 years	152	44.3
	5–10 years	49	14.3
	More than 10 years	35	10.2
Total		343	100.0

### Measures

This study used four instruments that were previously validated. They were translated using the back-translation procedure and were piloted with HR managers in each office who were not part of this study. A five-point Likert scale (1 = strongly disagree, 5 = strongly agree) anchored the items.

#### Psychological empowerment

To measure psychological empowerment, this study used a 12-item scale developed by Spreitzer (1995): competence, impact, meaning and self-determination. In the existing literature, acceptable estimates of reliability have been shown (Dust et al., 2014). In this study, the reliability coefficient was 0.89. An example question is “I have significant autonomy in determining how I do my job.” A confirmatory factor analysis (CFA) assuming the second-order factor indicated good data-model fit (*χ*^2^/df = 2.68, CFI = 0.94, TLI = 0.93, RMSEA = 0.05) with strong item factor loadings, ranging from 0.75 to 0.81.

#### Learning orientation

To measure learning orientation, an 11-item scale developed by Sinkula et al. (1997) was used. This scale consists of three sub-constructs: an organization’s commitment to learning, shared vision, and open-mindedness. In this study, internal consistency for this measure (Cronbach’s alpha) ranged from 0.88 to 0.90. An example question is “The basic values of this business unit include learning as key to improvement.” CFA suggested that the second-order factor model fit the data well (*χ*^2^/df = 3.69, CFI = 0.92, TLI = 0.91, RMSEA =0.06) with all items loading significantly on their corresponding factors (loadings range = 0.78 to.89).

#### Job embeddedness

Job embeddedness was assessed with a 7-item scale of global job embeddedness (Crossley et al., 2007) that was revised from composite job embeddedness developed by Mitchell et al. (2001). In previous studies, internal consistency for this measure (Cronbach’s alpha) ranged from 0.83 to 0.86 (Mitchell et al., 2001). Reliability scores in this study ranged from 0.88 to 0.90. An example question is “I feel attached to this organization.” CFA indicated reasonable data fit for the three-factor model (*χ*^2^/df = 1.27, CFI = 0.93, TLI = 0.91, RMSEA =0.05), ranging factor loadings of.78 to.85.

#### Affective organizational commitment

To measure affective organizational commitment, a 6-item scale developed by Meyer and Allen (1997) was used. This study’s reliability coefficient was 0.82. An example question is “I would be very happy to spend the rest of my career with this organization.”

### Control variable

The questions on demographic data consisted of a nominal scale, with male set at 1 female at 2, age in 20s at 1, 30s at 2, 40s at 3, 50s or older at 4. The educational background was set to high school graduates, bachelor, and graduate. The position was set to staff 1, assistant manage 2, general manager 3, director 4.

### Analytical approach

To examine the causal relationships among variables, this study employed structural equation modeling (SEM), which is quantitative research technique accounting for measurement errors (Kline, 2015). Two steps of data analyses were employed: (1) general assumption assessment including data distribution, reliability testing for measurement items, and validity testing for measurement structures as basic assessments for further data analysis, and (2) examinations of structural modeling on mediation analyses. First, basic assumptions for overall data analyses were tested (Hair et al., 2018). During this stage, according to the nature of research constructs, inter-construct correlation coefficient estimates were examined along with item internal consistency with Cronbach’s alpha coefficient estimates. In addition, CFA was performed to establish a valid measurement structure based on mode-data fit indices (Kline, 2015). Exploratory factor analysis was not considered, as all research constructs were validated and examined in previous studies across various contexts. Moreover, CFA results supported a sound level of construct validity for the proposed model (Hair et al., 2018). Second, to test hypotheses described in research framework, SEM analysis was performed to assess the direct and indirect effects between exogenous variables and endogenous variables (Kline, 2015). To examine structural equation modeling, we used AMOS 27.0 and SPSS 27.0.

## Results

Table 2 summarized results from descriptive statistics, zero-order correlations, and reliability coefficients for each of the study variables. The relationships of four variables are inter-correlated positively and significantly, revealing that multicollinearity is not a concern and that their inter-relationships require further analyses.

**Table 2:** Correlations and descriptive statistics (*N* = 343).

	Mean	SD	CR	AVE	1	2	3	4	5	6	7	8
1. Gender	1.27	0.45	–	–								
2. Age	2.15	0.88	–	–	−0.39^*^							
3. Education	1.92	0.06	–	–	0.21^*^	−0.23^**^						
4. Position	2.82	1.05	–	–	−0.22^*^	0.54^**^	−0.04					
5. PE	3.57	0.69	0.88	0.65	−0.09	0.19^**^	0.03	0.28^**^	(0.88)			
6. LO	3.38	0.59	0.84	0.64	−0.05	0.05	0.02	0.17^**^	0.32^**^	(0.83)		
7. JE	3.49	0.64	0.87	0.51	−0.06	0.09	0.01	0.12^**^	0.56^**^	0.43^**^	(0.88)	
8. OC	3.19	0.56	0.89	0.58	−0.13^*^	0.15^**^	−0.02	0.14^**^	0.49^**^	0.40^**^	0.67^**^	(0.89)

* *p* < 0.05;

** *p* < 0.01.

SD, Standard deviation; CR, Composite reliability; AVE, Average variance extracted; Cronbach’s alphas are shown in parentheses.

### Measurement model

**Figure 1:**
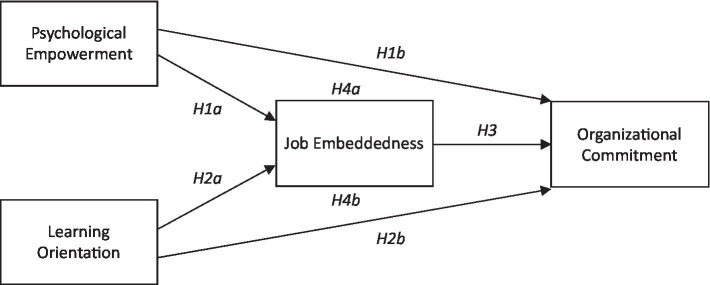
Research model with hypotheses.

This study employed confirmative factor analysis to examine the stability and validity of the proposed model. Fit statistics of the measurement model are as follows: *χ*^2^/df = 1.82, CFI = 0.94, TLI = 0.91, RMSEA =0.04. According to Hair et al. (2018), these fit indices revealed adequate model fit. Also, we examined the phi, correlations among the exogenous variables to further understand the extent to which a construct is truly distinct from other constructs. Results showed that discriminate validity existed among constructs. Convergent validity aims to understand the degree to which measures of the same concept are correlated. According to standardized *λ* and *T* values showed in Figure 1, latent variables reached a significant level, which represents the fact that every construct showed convergent validity.

Since this study relied on data assessed *via* employee self-reports, the possibility of common method bias (CMB) was checked. The Harman single factor test yielded four factors with eigenvalues greater than one that accounted for 72% of the total variance (Podsakoff et al., 2003). The first factor accounted for 26%, which is well below half of the total variance. Additionally, alternative models were compared. No other models improved the fit, less than.02 in the fit index. Consequently, the proposed model was adopted as the final model.

### Hypothesis testing

**Figure 2:**
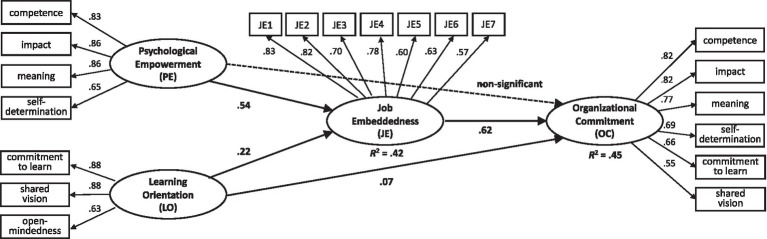
Results of structural estimates model analysis. *p* < 0.05.

Figure 2 indicates that psychological empowerment is positively related to job embeddedness (*γ* = 0.54, *p* < 0.01). Thus, Hypothesis 1a is supported. Consistent with Hypothesis 1a, learning orientation is also positively associated with job embeddedness (*γ* = 0.22, *p* < 0.01), thereby supporting Hypothesis 2a. Hypothesis 1b predicted that psychological empowerment is positively related to organizational commitment. However, the path coefficient is not statistically significant (*γ* = 0.05, *p* > 0.05). Thereby, Hypothesis 1b is not supported. Psychological empowerment does not have a direct effect on organizational commitment. In contrast, learning orientation is directly associated with organizational commitment. The path coefficient is statistically significant (*γ* = 0.07, *p* < 0.05). Therefore, Hypothesis 2b is supported.

To investigate the mediating effect of job embeddedness, this study examined the direct and indirect effect of structural and competing models. Path coefficients of the structural and competing models are represented in Table 3. The path between empowerment and job embeddedness, and the path between learning orientation and job embeddedness, were significantly related; however, their relationships with commitment were not significant. In addition to significant relationship between job embeddedness and organizational commitment, we observed that job embeddedness indirectly influenced the relationship with organizational commitment. Further, we examined the direct and indirect effects of the structural model. In Table 3, the influences of psychological empowerment on organizational commitment exist only in the indirect relationship. The indirect effect of job embeddedness is approved, and hypotheses 3 and 4 (both a and b) are supported as follows:

**Table 3:** Direct and indirect effects in structural and competing models.

Hypotheses	Coefficient	CR (*t* value)	Value of *p*	Result
Direct effect				
*1a*. PE → JE	*γ* = 0.540	8.480^**^	0.000	Supported
*1b*. PE → OC	*γ* = 0.045	1.254	0.210	Not supported
*2a*. LO → JE	*γ* = 0.216	4.072^**^	0.000	Supported
*2b*. LO → OC	*γ* = 0.065	2.126^*^	0.034	Supported
*3*. JE → OC	*β* = 0.615	11.072^**^	0.000	Supported
Indirect effect				
*4a*. PE → JE → OC^†^	*β* = 0.332	6.591^**^	0.000	Supported
*4b*. LO → JE → OC^††^	*β* = 0.133	6.095^**^	0.000	Supported

** *p* < 0.01;

* *p* < 0.05.

† Sobel test statistic: 6.768^**^.

†† Sobel test statistic: 3.833^**^.

Critical coefficient (*t* value) < 1.96 indicates nonsignificant relationships.

## Discussion

We investigated the role of job embeddedness on organizational commitment by assuming both a direct and an indirect effect of psychological empowerment and organizational learning orientation. Our results confirmed that all hypothesized relationships (PE and JE, LO and JE, LO and OC, JE and OC, and the mediating role of JE) are supported, except for psychological empowerment and organizational commitment. Aligned with previous literature, psychological empowerment was positively related to job embeddedness, especially considering the importance of psychological recourses on job embeddedness (Harunavamwe et al., 2020). However, psychological empowerment was not significantly related to organizational commitment in our present model. There may be a possible explanation that, depending on organizational culture or countries, the level of psychological empowerment and organizational commitment may be different (Jordan et al., 2017). In addition, Laschinger et al. (2002) suggested that the relationship between psychological empowerment and continuous commitment is low because continuous commitment, one of the organizational commitment variables, is related to the cost of leaving. Likewise, since the subject of the survey was small and medium-sized enterprises, it is analyzed that the relationship between continuous commitment related to leaving costs or external economic conditions may have played a greater role than affective commitment due to psychological empowerment.

Spector (1994) suggested that employees who feel high empowerment show a high degree of commitment to the organization. In addition, studies have been suggested that the higher the autonomy within the organization, the higher the job satisfaction and work efficiency (Seibert et al., 2004). Other studies reported psychological empowerment as a significant antecedent of organizational commitment (Joo and Shim, 2010; Ouyang et al., 2015). Bani et al. (2014) studied the association between psychological empowerment (in terms of sense of efficacy, meaningfulness, autonomy, and trust) and job embeddedness, and they found a positive association between those two constructs. Positive associations between psychological empowerment and job embeddedness were supported from several other studies (Jeon and Yom, 2014; Karavardar, 2014; Bin Jomah, 2017). In this study, while the impact of psychological empowerment was not significantly related to organizational commitment, it is notable that through job embeddedness, psychological empowerment had indirect effects on organizational commitment.

Further, learning orientation had significant effects on job embeddedness and organizational commitment. Findings of this study emphasize the role of learning organizations because learning orientation can support the ideas of employees’ psychological empowerment, organizational commitment, and job embeddedness (Islam et al., 2016). Learning organizations focus on adapting and generating new ideas with the belief that employees can continually learn how to work together and increase their capacity to create the results (Garvin et al., 2008). Aligned with the previous literature, learning organizations can act to be proactive rather than simply reactive to circumstances (Senge, 2006). This is accomplished by incorporating inputs from all levels of employees rather than receiving comments only from top management. This study can be useful for future researchers because not many researchers have used learning orientation as a predictor of job embeddedness, even if factors related to organizational culture were stressed in previous studies (Shah et al., 2020).

Findings from this study extend empirical literature on the positive effect of job embeddedness on organizational outcomes. The positive impact of job embeddedness has been examined numerously starting with reduced turnover (e.g., Lee et al., 2004; Shah et al., 2020), and expanded into other outcomes including job satisfaction and job performance (e.g., Halbesleben and Wheeler, 2008; Sun T. et al., 2012). These studies sequaciously supported job embeddedness as a significant mediator between individual characteristics or work context and individual’s psychological attachment. Although job embeddedness has been reported as a significant moderator between leadership and job performance (Sekiguchi et al., 2008), more scholars adopted the concept as an indicator of work efforts and energy that lead to positive organizational outcomes (Wheeler et al., 2012). Result of the significant indirect effect of job embeddedness mediating the relationship of psychological empowerment and learning orientation toward organizational commitment should prove to be a strong contribution to the growing body of knowledge and interests in employees’ affect toward their organization.

Lastly, the most compelling finding is a full mediation of job embeddedness in the relationship between psychological empowerment and organization commitment. That is, this result points to a potential job embeddedness-based mediator that adds to the organizational behavior mechanisms explored in past research. It shows that the job embeddedness only partially mediated learning orientation relationships; completely mediated the relationship in psychological empowerment and organizational commitment. Even if the direct effect of psychological empowerment on organizational commitment was not significant, we found that the indirect effect through job embeddedness was significant (Sobel test statistic: 6.768, *p* < 0.01). This finding indicates that employees’ job embeddedness plays a critical mediating role for employees to become more committed to work under conditions where employees are psychologically empowered and to work under a learning organization culture, which has not been examined in previous studies. Without job embeddedness of employees, even if employees are psychologically empowered or are exposed to a learning culture, employees may not commit themselves to organizational activities. This finding highlights ongoing interactional networks of social relations in critical awareness and problem-solving, and how well the work environment suits employees. People guide their commitment based on social interactions with peers and continue to deal with those they trust (Chan et al., 2008).

### Implications for practice and research

Our study has several implications for practice and research. The presented structural model may be adopted as a reference tool for practitioners when addressing improvements for the awareness of organizational jobs and commitment. First, this study suggests that programs targeted toward enhancing organizational commitment may focus on the concept of job embeddedness and include psychological empowerment and learning orientation as focal points. Ultimately, job embeddedness is a psychometrically sound construct that captures employees’ work energy and efforts that help them to understand meaning, importance, and sustainment relative to their job. Effectiveness and promise of employee assistance programs’ improving employees’ job embeddedness related to lowering turnover has been well documented in the literature (Wheeler et al., 2007). Investments in empowerment training is often questioned and compared against a single outcome, such as employee’s commitment and well-being. When their effect on what job embeddedness is accounted for, leaders and managers will better understand the role and efficacy of employee empowerment that may instill greater attachment to working groups, encourage their motivation, and lead to greater commitment (Sun L. Y. et al., 2012).

Also, scholars can build upon our model to further expand research on the subject. Researchers can re-examine this suggested structural equation model by replacing the existing variables with other cognate variables. For example, Dweck et al. (2014) included a growth mindset variable instead of learning orientation, which we adopted to capture the employee’s motivational state as a response to the organization. Future researchers can also seek other environmental factors that help to create a learning organization. Other factors, such as organizational climate, managerial support, and a psychologically safe environment can be further included in the structural model as exogenous or indirect effects to continuously update and expand the body of relevant research.

Particularly, scholars can add the shared vision of an organization and its acceptance to the organizational members into the model, given the rising interest of the match between organizational values and important outcomes of society and customers. A shared vision among employees helps to provide focus for the organization as a whole, allowing for momentum and drive towards a vision through job embeddedness. This vision is different from an individual vision in that it is more important for the whole to possess and understand the vision than it is for any individual; it is something that binds individuals together (Senge, 2006).

### Limitations and future research suggestions

Some limitations need to be recognized. First, the generalizability of the results should consider the sampling. Although data were collected from multiple industries, participants were those who attended Human Resource Development training in South Korea. We need to examine if the sample of employees without training has similar patterns or not. In addition, studies conducted across different nations and continents tend to enrich the validation of a proposed model. Second, this study focused on the effects of each variable based on one-time data collection. Exploring the effects of the model based on time gaps, especially considering the time needed to transfer employee assistance interventions will be particularly helpful. Relatively few studies have used longitudinal data to study job embeddedness (Gallie et al., 2017).

Third, a self-reported instrument was used, which may be subject to respondent biases, such as the inability to provide accurate responses because of insufficient recall or memory. Also, a CMB (Podsakoff et al., 2003) is a greater challenge in a cross-sectional study. Although various measures were applied, such as single factor and alternative models testing, as well as an examination of convergent and divergent validity, other useful techniques, such as a marker variable testing, exist. As with all other times when using the same Likert-type scale, the variance that the scales shared with each other represent a response bias. There may be central tendency bias and social desirability bias, which are common for any Likert-type scale.

In conclusion, results of this study suggest that employee’s on-and off-the job causes of turnover may enrich knowledge of commitment, increasing it beyond the current focus on employee’s retention. Psychological empowerment and learning orientation were significantly predictive of organizational commitment through job embeddedness. This broader impact of job embeddedness extends theory and suggests compelling directions for future study

## Data availability statement

The original contributions presented in the study are included in the article/supplementary material, further inquiries can be directed to the corresponding author.

## Author contributions

DY and CH contributed to conception and design of the study. SL and MS organized the database and performed the statistical analysis, wrote the first draft of the manuscript. JC and SH wrote sections of the manuscript. All authors contributed to the article and approved the submitted version.

## Funding

We would like to thank that this paper was supported by Konkuk University in 2019.

## Conflict of interest

The authors declare that the research was conducted in the absence of any commercial or financial relationships that could be construed as a potential conflict of interest.

## Publisher’s note

All claims expressed in this article are solely those of the authors and do not necessarily represent those of their affiliated organizations, or those of the publisher, the editors and the reviewers. Any product that may be evaluated in this article, or claim that may be made by its manufacturer, is not guaranteed or endorsed by the publisher.
